# A Rare Occurrence of Multiple Intracranial Osteomas in the Cranial Cavity of a Cadaver With a Short Review on Subdural Osteoma

**DOI:** 10.7759/cureus.17737

**Published:** 2021-09-05

**Authors:** Arundhati Kar, Manisha Gaikwad, Madhumita Patnaik

**Affiliations:** 1 Anatomy, All India Institute of Medical Sciences, Bhubaneswar, IND

**Keywords:** osteomas, falx cerebri, tentorium cerebelli, intracranial, new classification

## Abstract

Osteomas are most common among all primary bone tumors of skull bones. They are usually asymptomatic due to their small size and slow growth. They are found incidentally on imaging studies for other neurologic symptoms. Osteoma may be single or multiple when present. They should be differentiated from meningiomas, chordomas, schwannomas, and parosteal osteosarcoma by using different diagnostic methods, including histopathologic study.

During routine dissection for MBBS students in an 87 years old female cadaver, we found multiple (seven in number) irregular, lobulated bony masses/structures. Their positions were different with respect to the layers of meninges. Some were present between the dura mater and arachnoid mater compressing the adjacent brain tissues forming impressions on them, and some were outside the dura mater. So, into the previously existing classification, we want to add a new variety under the type b category, i.e., mixed type (intraparenchymal, dural, skull vault) as pointed under the subtype V, which is found in our case.

## Introduction

Osteomas are small trabeculated bones present either intracranially or extracranially. They are most common among all primary bone tumors of skull bones [1]. Usually, they are asymptomatic if present due to their small size and slow growth. They are found incidentally either in computed tomography (CT) or magnetic resonance imaging (MRI), done as an investigation for other neurologic symptoms. Osteoma may be single or multiple when present. In the present case, osteomas are found incidentally during routine cadaveric dissection.

There are many theories supporting its etiology, such as traumatic, infectious, and developmental causes, but no theory is conclusive yet. Some theories include post-trauma or post sinusitis inflammatory response increasing the osteoblastic activity of mucoperiosteum in paranasal sinuses and causing mature bone formation [2]. Cranial vault osteomas are usually asymptomatic. The embryological origin of osteomas is thought to occur from periosteal cells or embryologic cartilage cells near the cranial vault bones [3,4].

Clinically, the osteoma and parosteal osteosarcomas show similar pressure features like headache, intermittent dizziness, altered mental status depending on their size and the location related to the brain. But osteosarcoma has a worse prognosis as it is malignant and needs postoperative adjuvant treatment in contrast to benign osteoma. Therefore, they are differentiated by histopathological study. Osteoma shows mature lamellar bone in trabeculae with fibrovascular stroma surrounded by osteoblasts. But, long-standing osteomas may lose this osteoblastic activity [5]. On MRI scan, the differential diagnosis for osteomas are meningiomas, chordomas, and schwannomas. So, recently 3D CT reconstruction is used for its accurate localization and morphology [5].

## Case presentation

During routine dissection practical for MBBS students in an approximately 87 years old female cadaver, we found multiple (seven in number) irregular, lobulated bony masses/structures at different locations in the cranial cavity. Three of them were between the dura mater and arachnoid mater compressing the adjacent brain tissues forming impressions on them, and the rest four were outside the dura mater. No osteomas were found extracranially or in other parts of the body of the reported cadaver.

**Figure 1:**
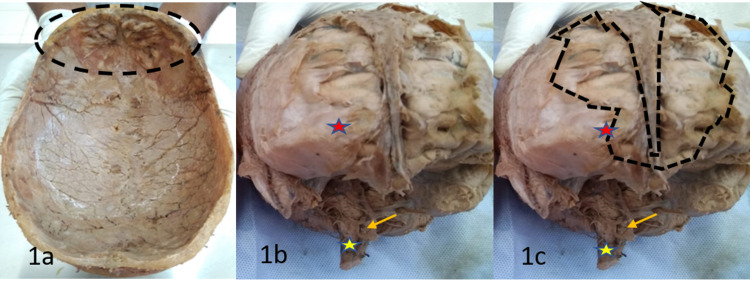
(a) The skull cap was cut to teach the students, location of the dural venous sinuses. On examination, the calvaria in the frontal region showed elevated bony ridges, which were tightly connected to the dura adjoining it. (b) Red star mark showing the frontal pole, yellow star mark showing the brain stem, and yellow arrow mark showing the basilar artery. (c) Dotted line showing the dural tear and frontal lobe impression.

Dura mater was intact in all the areas where bony masses were noted, except one place, i.e., just in front of the frontal pole (Figures 1a-1c). At this area, the bony mass was adherent compactly to the inner table of the skull so that the dura mater was torn off while taking out the brain from the cranial cavity. It was the largest bony mass among all. This bony mass must have formed from the subdural space. It adhered tightly to the inner table of the skull. So, it was inseparable from the skull cap.

The other six bony masses were separated from the dura mater easily. They were irregular in shape. So their maximum dimensions in each axis (i.e., X, Y, and Z-axis) were measured using the digital vernier’s calliper (Digimatic Callipers of Yamayo classic). Only for the biggest mass, dimension was measured in two axes (i.e., in the X and Y-axis). Its maximum thickness was measured, including the outer table from which the skull vault thickness was subtracted manually to get its approximate thickness. The dimension of all the bony masses was recorded in Table 1.

**Table 1:** Dimension of all the masses and their location in dura mater

Bone number	Location	Dimension measured in mm^3^
1	Inner table of the skull	41.41 × 24.04 × 6.41
2	The left side of falx cerebri	28.21 × 9.67 × 4.26
3	Near right pterion	24.72 × 10.23 × 3.25
4	Near right asterion	13.58 × 6.32 × 2.8
5	The upper surface of the right tentorium cerebelli	9.72 × 8.69 × 3.41
6	Left anterior cranial fossa	11.86 × 6.80 × 2.37
7	The upper surface of the left tentorium cerebelli	10.10 × 7.41 × 2.81

**Figure 2:**
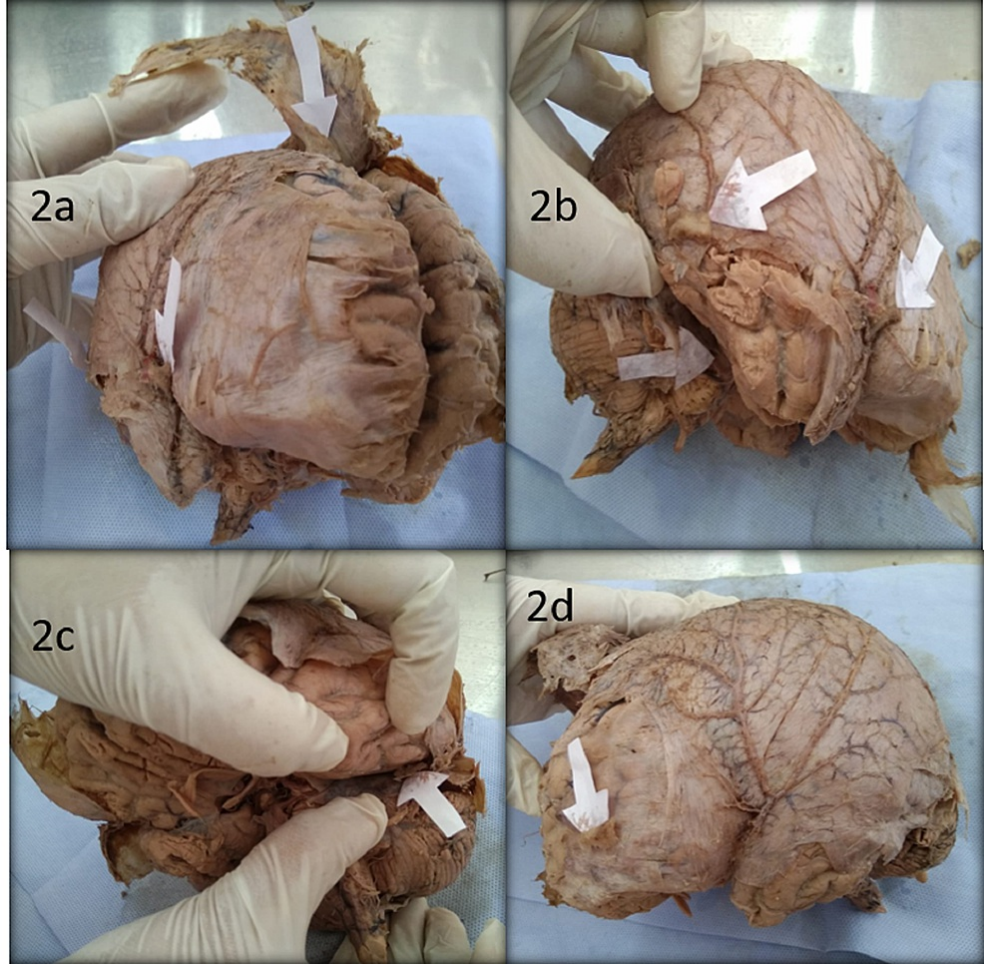
White paper arrow marks showing the intact bony masses and their locations - (a) at the left side of falx cerebri, (b) at the upper surface of right tentorium cerebelli near right asterion, near right pterion (from left to right), (c) the upper surface of left tentorium cerebelli, and (d) left anterior cranial fossa.

The locations of six small bony masses (Figures 2a-2d) were recorded. The largest detachable bony mass (dimension - 28.21 x 9.67 x 4.26 mm^3^) was nearer to the inferior sagittal sinus on the left side, approximately 5 cm behind the frontal pole. It was between the meningeal layer of the dura mater and the arachnoid mater. They were also present in other locations with their flat surface toward the meningeal layer of the dura mater and slightly adherent to it through a few fibrous tissues. The histological section after decalcification and routine hematoxylin and eosin staining of the bony masses was taken and observed under the microscope.

Microscopic features

**Figure 3:**
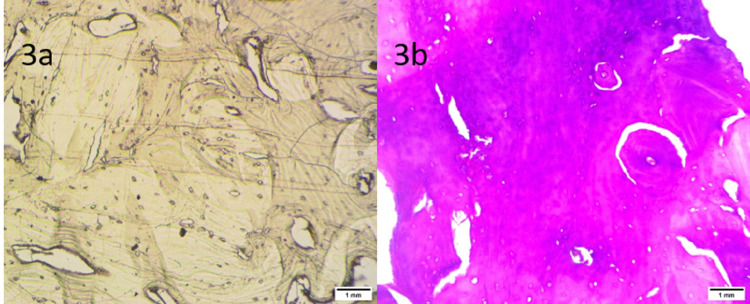
Unstained (a) and stained with H&E (b) slides of decalcified bones at 10x magnification.

On the histopathologic study of the decalcified mass, osteocytes were found in lamella forming the central part and scattered at the peripheral region. The lamellated bony trabeculae and the intertrabecular spaces were occupied by a scanty amount of loose fibrovascular tissue. Figures 3a, 3b showed the unstained (left) and stained (right) slides of decalcified bones at 10x magnification.

## Discussion

The occurrence of intracranial osteomas is extremely rare, usually single in number, and mostly develops along the inner table of skull bone or the convexity of meninges [5]. Among them, only a small percentage of cases show origin from falx and no case from tentorium. The classification of osteomas till now is as follows according to different authors (except type b - subtype v). The first type depends on the proportion of dense and cancellous bone: i) ivory (dense, mature, lamellar bone with little fibrous stroma) and ii) mature (large trabeculae of mature, lamellar bone with more abundant fibrous stroma and may or may not have osteoblastic rimming) [1].

The second classification is depending on the origin and location. i) intraparenchymal (no connection to dura or bone, rarest type); ii) dural (no bony attachment, arise mainly from the falx, are asymptomatic and are often incidental findings on plain radiographs); iii) skull base (most common in the frontal sinus but may also occur in the ethmoid air cells, maxillary and sphenoid sinuses); iv) skull vault - may arise from the outer table (exostotic) or inner table (enostotic), and are usually asymptomatic; and v) mixed type (intraparenchymal, dural, skull vault) [6].

In histological fields, the multinucleated giant cells explain the infectious reaction, and the fibro-osseous lesion explains no tendency to metastasize [7].

For the present case, we postulate that, as there is a scant or minimal connective tissue stroma, it is a case of multiple very mature intracranial osteomas (ivory variety according to the classification type a) [1]. But, this case does not fit into any of the subtypes of this existing classification in type b. So, we have added a new variety under the type b category, i.e., can be pointed under the subtype V, i.e., mixed type (intraparenchymal, dural, skull vault), which is found in our case (not reported previously).

All the eight reported subdural cases with clinical features [8-15] are discussed in Table 2. Out of these, most cases presented with age more than 50 years, including the present study, except three cases [9,10,14]. The expected association of trauma was seen with only one case [13], whereas others did not have any associated suggestive history.

**Table 2:** All the cases of subdural osteomas in falx cerebri and the age of symptoms presentation with associated clinical and microscopic findings

Year	Literature	Age/sex	History	Histology
1995	Choudhury [9]	20-year-old woman	Four-month history of persistent right frontotemporal headache	Thick bony trabeculae enclosing marrow spaces
1998	Aoki [8]	51-year-old woman	A 10-year history of headache	Dense laminar cortical bone containing marrow spaces occupied by adipose tissue
2002	Cheon [10]	43-year-old female	Two-year history of constant headache	Mature lamella bone, made of Haver’s system, and normal osteocytes between osteoid layers
2007	Jung [11]	60-year-old man	Three-year history of persistent headache	Lamellated bony trabeculae lined by osteoblasts
2012	Barajas Jr [12]	63-year-old woman	Altered mental status	The mature lamellar bone that displayed an irregular interface with adjacent gliotic brain parenchyma
2016	Cao [13]	54‑year‑old male	Five‑month history of intermit­tent dizziness	Lamellated bony trabeculae lined with osteoblasts and the intertrabecular marrow spaces were occupied by adipose tissue having no active osteoblastic or osteoclastic activity
2016	Kim [14]	29-year-old female	Three-year history of headaches	Fatty marrow within the ma­ture trabecular bone
2018	Yang et al. [15]	64‑year‑old female	Intermittent dizziness. No history of head trauma or infection.	The lesion predominantly consisted of lamellar bone without bone marrow elements
2020	Our Present study	87-year-old female	Unknown symptom and cause Multiple sources (intraparenchymal, dural, and from skull vault)	Osteocytes in lamella at the center and scattered at the periphery. So osteons may be spherical or cylindrical in shape. Bone marrow was not evidenced in any of the histological slides. The lamellated bony trabeculae and the intertrabecular spaces are occupied by a scanty amount of loose fibrovascular tissue. So, osteons are secondary osteons.

Most of them presented with headaches except three, out of which two presented with intermittent dizziness [13,15] and one with progressive altered mental status [12]. In the cases of headache, the meningioma was found to be at the anterior cranial fossa (either right or left side). Among the cases with intermittent dizziness, in one case, the mass was beneath the right parietal bone [13] whereas, in the other, it was in the middle cranial fossa [15] (exact location not mentioned). In the case showing altered mental status, the mass measuring 4.5 × 3.7 × 2.5 cm was located on the right side; the middle cranial fossa had a mass effect upon the right-side temporal lobe [12]. Hence, the varying symptoms may depend on the location related to the brain.

The localization in most cases of subdural osteoma could be done by using CT, whereas, for proper diagnosis, they all had done MRI scans. For one case, [10] intraoperative radiography was essential to identify the cause.

Symptoms in the present case (87 years old female) are unknown as it was a donated cadaver, though the osteomas are arising from different sources. The largest one is expected to be arising from the frontal dura mater and others from falx cerebri, tentorium, and parietal dura mater. As per our knowledge, no case has been reported with tentorial osteoma or osteomas of multiple origins. So, the expected clinical presentation is not known. However, it can be postulated that there will be a mass effect on the occipital lobe and the cerebellum if the size of the tentorial mass is more extensive. Hence, this case appears to be a rare variety of multiple mature intracranial osteomas with a different source of origin. This is the novelty of this case. As there is an increase in the size of the mass, the patient would likely have presented with generalized intracranial pressure effects like nausea, headache, seizure, psychosis, etc., or localized pressure effect, according to the location and size of the lesion [8,16].

## Conclusions

Among all types of intracranial osteomas, subdural osteomas are rarest of all types. The present case is presenting with the eldest (87 years) of all subdural osteoma cases. Also, it has been associated with intradural as well as skull vault osteoma.

Whenever subdural osteomas are present, mostly, they are multiple. If there is an increase in the size of the mass, the patient will usually present with generalized intracranial pressure effects like nausea, headache, seizure, psychosis, etc., or localized pressure effect, according to the location and size of the lesion. Also, incomplete removal will lead to recurrence. So, it is essential to identify and remove other subdural osteomas if one is identified on CT or MRI as they do not tend to metastasize. Complete surgical resection whenever possible seems to be the curative treatment.
